# Pre-coincidence brain activity predicts the perceptual outcome of streaming/bouncing motion display

**DOI:** 10.1038/s41598-017-08801-5

**Published:** 2017-08-18

**Authors:** Song Zhao, Yajie Wang, Lina Jia, Chengzhi Feng, Yu Liao, Wenfeng Feng

**Affiliations:** 1 Department of Psychology, School of Education, SooChow University, Suzhou, Jiangsu 215123 China; 2 Department of Education, School of Humanities, Jiang Nan University, Wuxi, 214122 China

## Abstract

When two identical visual discs move toward each other on a two-dimensional visual display, they can be perceived as either “streaming through” or “bouncing off” each other after their coincidence. Previous studies have observed a strong bias toward the streaming percept. Additionally, the incidence of the bouncing percept in this ambiguous display could be increased by various factors, such as a brief sound at the moment of coincidence and a momentary pause of the two discs. The streaming/bouncing bistable motion phenomenon has been studied intensively since its discovery. However, little is known regarding the neural basis underling the perceptual ambiguity in the classic version of the streaming/bouncing motion display. The present study investigated the neural basis of the perception disambiguating underling the processing of the streaming/bouncing bistable motion display using event-related potential (ERP) recordings. Surprisingly, the amplitude of frontal central P2 (220–260 ms) that was elicited by the moving discs ~200 ms before the coincidence of the two discs was observed to be predictive of subsequent streaming or bouncing percept. A larger P2 amplitude was observed for streaming percept than the bouncing percept. These findings suggest that the streaming/bouncing bistable perception may have been disambiguated unconsciously ~200 ms before the coincidence of the two discs.

## Introduction

The human visual system often experiences unitary and stable perception by integrating relevant sensory information in the environment. However, on occasions when the available sensory information is fragmented or ambiguous, our perception may alternate between two or more mutually exclusive interpretations^1^. Well-known examples of this bistable perception include the famous face-vase drawing, Necker cube and binocular rivalry. Additionally, bistable perception occurs not only in the static figures mentioned above, but in moving objects as well. Consider two identical visual targets moving toward each other along the same horizontal line with equal and constant speed in a two-dimensional display: Two visual targets start their motion from opposite sides, coincide at the center of the screen, move apart, and stop at each other’s starting point. In this display, observers typically perceive the motion of the targets after coincidence as either “streaming through” or “bouncing off” each other^2^.

Despite the ambiguous nature of the streaming/bouncing motion display, subjects usually show a strong bias to report the streaming percept (~80% streaming response vs. ~20% bouncing response)^3–5^. Interestingly, a large number of behavioral studies have reported various factors that can reverse perceptual dominance from streaming toward bouncing, such as a momentary pause of the two discs^3, 5^, a brief sound^6–14^, a transient visual distractor^4, 15^, and even a transient tactile vibration^16^ at the moment of coincidence of the two discs as well as post-coincidence trajectory duration^17, 18^, pre-coincidence trajectory switches (used to manipulate expectation)^19^, a partial overlap^20, 21^ of the two discs, and an orientation difference between the stripes on the discs’ and their path of motion^15^. Several neuroscience studies have explored the neural mechanisms of the effect of sound on streaming/bouncing motion display (i.e. the audiovisual bounce-inducing effect, ABE). For example, an event-related functional magnetic resonance imaging (fMRI) study has shown that a transient sound induced higher activation in multimodal areas (e.g., the prefrontal and posterior parietal cortex) when subjects perceived bouncing than streaming^22^. An electroencephalograph (EEG) study found increased beta-rhythm synchronization across frontal, parietal, and occipital cortex and gamma-rhythm synchronization across central and temporal regions for bouncing trials compared to streaming trials when the coincident sound was introduced^23^. Furthermore, using transcranial magnetic stimulation (TMS) to temporarily deactivate the posterior parietal cortex resulted in an attenuated magnitude of the sound’s effect on improving the bouncing percept^24^. Combining these neuroscience studies, cross-modal integration between polysensory and unisensory brain cortices may underlie the effect of sound on reversing the perceptual dominance of the streaming/bouncing motion display.

Despite numerous identified bounce-inducing factors, especially evidences based on neural mechanisms for the audiovisual bounce-inducing effect (ABE), the neural basis of disambiguating perception during the streaming/bouncing bistable perception processing in purely visual streaming/bouncing display remains unclear. In other words, it is not understood how one percept (streaming or bouncing) overwhelms the other and eventually becomes a stable percept under an ambiguous context in a visual streaming/bouncing bistable display. Sekuler and Sekuler as well as Watanabe and Shimojo have proposed that the human perceptual system might utilize experience of the three-dimensional world in a probabilistic way to derive a stable percept in a two-dimensional visual streaming/bouncing motion display^5, 7, 17^. For example, Watanabe and Shimojo argued that moving objects in a natural environment are seldom (but still possibly) aligned at the same depth plane^7, 17^. In other words, the bouncing event in a three-dimensional world would occur only when the two moving balls exist in the same depth plane (it’s less possible but still occurs occasionally). Our perceptual system may compute this probability when facing a visual streaming/bouncing motion display, so the streaming percept could be dominant and the visual streaming/bouncing motion display could potentially be ambiguous. Although this probabilistic inference account is highly reasonable and has been confirmed by several studies^13, 19, 20^, there is little experimental evidence to support this account in the context of a purely visual streaming/bouncing motion display^19, 25^.

In previous bistable perception studies, a traditional view proposed that antagonistic activity within the visual system is a neural consequence of spontaneous perceptual reversals^26, 27^. Consistent with this viewpoint, many studies have found neural activity in the primary visual cortex^28–32^, lateral geniculate nucleus^33, 34^, and extrastriate visual cortex^35–39^ that correlates with perceptual outcomes or perceptual transitions in ambiguous displays. However, a growing number of fMRI studies have demonstrated that the frontal and parietal cortex play a causal role in initiating perceptual reversals in bistable displays^37, 40–43^, which indicates that high-level brain areas could reorganize ambiguous information within the visual cortex and eventually reverse our perceptual interpretations^1, 44, 45^. On the other hand, a series of event-related potential (ERP) studies have identified two successive ERP components (difference wave) related to perceptual reversals of bistable stimuli, which are the occipital-parietal “reversal negativity” (RN)^46–51^ and the central-parietal “late positive complex” (LPC)^47–50, 52^. These phenomena were discovered by comparing trials on which perceptual outcome was reversed to trials on which the perceptual outcome remained the same across successive trials. The RN (170–300 ms post-stimulus) is considered to be an early index of switches between neural representations that synthesize the current contents of conscious perception, while the LPC (400–600 ms) is thought to represent post-perceptual processing related to evaluating and reporting perceptual outcomes^49, 53, 54^. Therefore, the emerging brain model of bistable perception is a dynamic and highly interactive network between low-level (sensory) and high-level (frontal and parietal) brain regions.

Apart from the reversal-related brain activities that are associated with the perception reversals mentioned above, the percept-related brain activities that are associated with the ongoing stable percept deriving should also be highlighted in bistable perceptions, especially in the case of visual streaming/bouncing display because “streaming through” and “bouncing off” of the two visual discs after coincidence are completely opposite perceptual states in nature. Therefore, exploring the neural basis of streaming and bouncing percepts in the visual streaming/bouncing display can contribute to better understandings of the issue of how and when one perceptual state prevails over the other and eventually becomes a stable percept during an ambiguous situation in bistable perceptions along with the well-known effect of sound on reversing the perceptual dominance of the streaming/bouncing display (ABE). Using high density ERP recordings, the present study investigated the brain dynamics of streaming/bouncing bistable motion processing. A trial-based analysis was performed to investigate neural activities in trials on which subjects reported streaming percepts (streaming trials) with trials on which bouncing percepts (bouncing trials) were reported. Surprisingly, the amplitude of the P2 component over the frontal central scalp ~200 ms before the coincidence of two discs was found to be larger for the steaming percept than for the bouncing percept. These results demonstrated that brain activity ~200 ms before the coincidence of two discs was predictive of the subsequent perceptual outcome of the visual streaming/bouncing display.

## Materials and Methods

### Participants

A total of 23 healthy subjects participated in this study after giving informed consent as required and approved by the Human Research Protections Program of SooChow University. All methods were carried out in accordance with the relevant guidelines and regulations. All participants had normal or corrected-to-normal vision and were naive to the purpose of the experiment. Data from 5 subjects were eliminated due to an inadequate number of trials (less than 60 trials) for one of the perception outcomes (streaming or bouncing), leaving data of 18 subjects (11 female, mean age of 21.6 years) for further analysis.

### Stimuli and procedure

**Figure 1:**
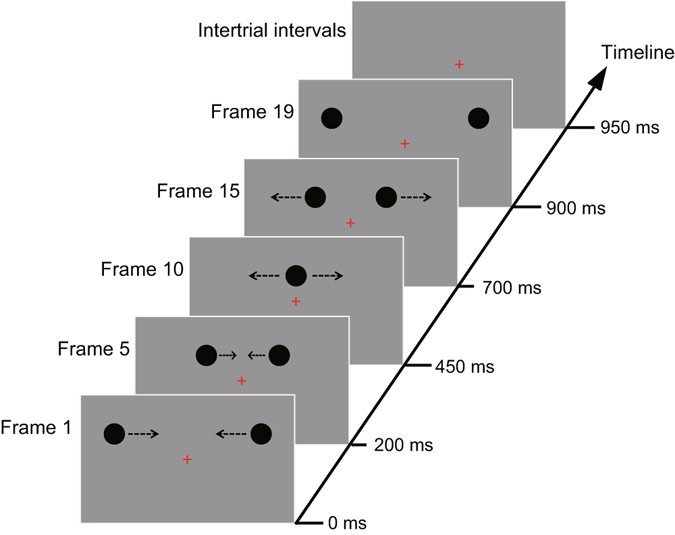
Schematic illustration of the visual streaming/bouncing motion display. Example shown is for a response-required trial in which two identical black discs started their motion from opposite sides, moved toward each other, coincided, moved apart, and stopped at each other’s starting points and then disappeared, followed by an intertrial interval (ITI) of 1200–1600 ms. The long solid axis (timeline) represents the onset moments of frames and each frame lasted for 50 ms. The dashed arrows near the discs indicate the direction of the motion of the discs in the next frame and are not presented in the experimental displays. On catch trials, the stimuli (except for the fixation cross) were absent from frame 10 through 19. Subjects were instructed to report their perceptual outcomes (streaming or bouncing) via a button-press on response-required trials and not respond on catch trials.

The experiment was conducted in a dimly lit, sound attenuated chamber. All stimuli were constructed and scripted using “Presentation” software (Neurobehavioral Systems, version 18.0) and were presented on a 27-inch LCD monitor (ASUS VG278HE, refresh rate 100 Hz, resolution 1920 × 1080). A small red cross (0.3° × 0.3° of visual angle) served as a fixation point and was presented at the center of the gray background (10 cd/m^2^ of luminance) throughout each block. In 66.7% of trials (response-required trials), two identical black discs (each 1.05° in diameter) were initially presented at the opposite edges of the background. The discs were separated by 18.9° (visual angle) horizontally and placed 3.46° above the fixation cross on the first frame (the duration for each frame was 50 ms). From frame 2 through 9, the two discs moved toward each other along same horizontal path. Each frame was presented immediately after the offset of the preceding frame (i.e., frame to frame SOA was 50 ms). On frame 10 (450 ms after the onset of the first frame), the two discs visually coincided above the fixation cross. From frame 11 through 19, the discs moved apart from each other and stopped at the starting point of the opposite disc (Fig. 1). Given the initial distance of 18.9° and the duration of 50 ms for each frame as well as a total of 19 frames, the two movement of the two discs took 900 ms with a constant speed of 21°/s (i.e., 1.05° per frame). In the other 33.3% of trials (“catch” trials), the stimuli presented on frame 1 to frame 9 were exactly the same as of the response-required trials. However, no stimulus was presented from frame 10 through 19 (i.e. frame 10 to frame 19 were presented as blank frames) on catch trials. In other words, the discs moved toward each other and then suddenly disappeared just before their coincidence, which produced neither a streaming nor bouncing percept. These catch trials were included in the experiment to ensure that subjects were responding veridically based on their perceptual outcomes after the coincidence event occurred and not simply based on guesswork before that event ocurred.

Subjects observed the stimuli display from a distance of 85 cm while holding their gaze on the fixation cross during the experiment. Their assigned task was to report whether the two discs appeared to be “streaming through” or “bouncing off” each other after coincidence based on their intuition during response-required trials by pressing one of two buttons on a keyboard. The response buttons for “streaming” and “bouncing” percepts were counterbalanced across participants. No responses were required when the motion of the two discs disappeared before coincidence on catch trials. Both types of trials occurred on each block with a given probability (response-required trials: 66.7%; catch trials: 33.3%) in a randomized sequence and the intertrial intervals (ITI) varied from 1200 to 1600 ms (Fig. 1). The whole experiment consisted of 15 blocks of 60 trials each and subjects were allowed to take a short break after finishing each block.

### Electrophysiological recordings and analysis

The electroencephalogram (EEG) was recorded continuously using a 64-channel tin-electrode cap (Quik-Cap, NeuroScan, Inc.) based on an extended 10–20 system montage. Standard 10–20 sites were FP1, FPz, FP2, F7, F3, Fz, F4, F8, T7, C3, Cz, C4, T8, P7, P3, Pz, P4, P8, O1, Oz and O2^55^. Additional intermediate sites were AF3, AF4, F5, F1, F2, F6, FC7, FC5, FC3, FC1, FCz, FC2, FC4, FC6, FC8, C5, C1, C2, C6, TP7, CP5, CP3, CP1, CPz, CP2, CP4, CP6, TP8, P5, P1, P2, P6, PO7, PO5, PO3, POz, PO4, PO6, PO8, CB1 and CB2. Horizontal eye movements were monitored by two bipolar electrodes at the left and right external canthi (horizontal EOG). Vertical eye movements and blinks were monitored via two bipolar electrodes above and below the left eye (vertical EOG). The left mastoid electrode served as the reference and all electrode impedances were kept below 5 kΩ during data acquisition. The raw EEG and EOG signals were amplified with a gain of 10,000, filtered with an amplifier bandpass of 0.05–100 Hz, and were digitized with a sampling rate of 1000 Hz. The EEG signals on response-required trials were averaged in 1200 ms epochs time-locked to the onset of the two visual discs (i.e. time-locked to the onset of frame 1, see Fig. 1) with a 200-ms pre-stimulus baseline. Epochs contaminated by eye movements, eye blinks, muscle activity, or amplifier blocking were discarded before averaging, leaving a total of 512 ± 11 valid epochs (mean ± SE) on response-required trials for averaging. The resulting averaged ERP waveforms were then digitally low-pass filtered (3 dB cutoff at 30 Hz) to remove high-frequency noise produced by muscle movements and external electrical sources. After filtering, all averaged ERP waveforms were then re-referenced to the algebraic average of the left and right mastoid electrodes.

To examine the neural activities of the streaming percept and bouncing percept, ERPs recorded on the response required trials were analyzed on the basis of the subjects’ perceptual responses. A trial-by-trial analysis was performed by separating the response required trials on which subjects reported streaming percept (streaming trials) from trials on which bouncing percept (bouncing trials) were reported. ERP waveforms were averaged separately for streaming trials and bouncing trials. On average, there were 315 ± 22 (mean ± SE) streaming trials and 197 ± 18 bouncing trials. The main ERP components in both streaming and bouncing trials were quantified as the mean amplitudes with respect to a 200-ms pre-stimulus baseline over the following time windows and with the following electrodes: (1) C1 component (75–95 ms, measured over POz, PO3, PO4, Oz, O1, O2); (2) P1 component (120–145 ms, measured over the same electrodes as the C1 component); (3) N1 component (170–200 ms, measured over the same electrodes as C1 component); (4) P2 component (220–260 ms, measured over FCz, FC1, FC2, Cz, C1, C2); (5) N400 component (350–440 ms, measured over the same electrodes as P2 component); (6) N600 component (560–660 ms, measured over the same electrodes as P2 component); and (7) P3 component (800–900 ms, measured over CPz, CP1, CP2, Pz, P1, P2). These time windows and their corresponding measurement electrodes were chosen because each ERP component had its maximal amplitude over its given time windows and electrodes. After quantifying these main ERP components, the mean amplitudes of each ERP component were then subject to a two-way repeated-measure ANOVA with factors of perceptual response (streaming/bouncing) and the electrode (channels listed above for each ERP component), respectively. When appropriate, ANOVA results were corrected using the Greenhouse-Geisser procedure. Post-hoc comparisons for the main effects of electrode were made to determine the significance of pair-wise contrasts when appropriate, using the Bonferroni adjustment for multiple comparisons.

## Results

### Behavioral results

The percentage of certain responses (streaming or bouncing), and reaction times (measured relatively to the moment of coincidence, i.e., the onset of frame 10) between streaming trials and bouncing trials were compared by ANOVA, respectively. Consistent with the findings in previous behavioral studies that streaming percept dominates in the original visual streaming/bouncing motion display^3–5^, the percentage of streaming responses in our study was significantly higher compared to the bouncing responses [streaming, 61.2 ± 3.6% (mean ± SE) of response required trials; bouncing, 38.8 ± 3.6% of response required trials; *F*(1, 17) = 9.58, *p* < 0.007, *η* ^2^_*p*_ = 0.36]. Although there was a trend of faster reaction times for streaming responses compared to bouncing responses (streaming, 524 ± 20 ms; bouncing, 538 ± 21 ms), which appears to be consistent with the observation that the dominant percept (i.e. streaming) in the visual streaming/bouncing display leads to shorter reaction times^10^, this difference did not reach significant level [*F*(1, 17) = 2.13, *p* = 0.163, *η* ^2^_*p*_ = 0.11] in the present study.

### ERP results

#### *Pre-coincidence P*2 *amplitudes predict streaming/bouncing percepts*

**Figure 2:**
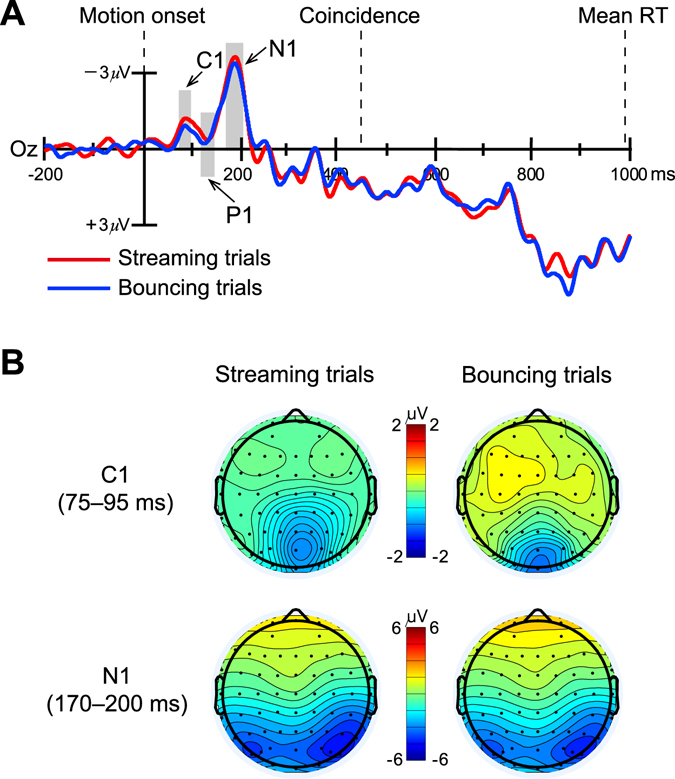
Grand-averaged ERPs associated with the streaming and bouncing percepts over the posterior scalp. (**A**) Three early visual-evoked ERPs over the occipital scalp, C1 (75–95 ms, shaded area, the same below), P1 (120–145 ms), and N1 component (170–200 ms), elicited by the visual streaming/bouncing motion display. Shaded areas show the time windows in which the three components were quantified respectively. Example waveforms shown here are from Oz electrode. Key events in behavioral task [motion onset, coincidence, and mean RTs of all response-required trials (measured relatively to the moment coincidence, i.e., the onset of frame 10)] are shown above the ERP waveforms. The dash line connecting the key events and ERP waveforms indicates they share same time axis in this figure. (**B**) Topographical distributions for the visual-evoked C1 (75–95 ms) and N1 (170–200 ms) components. Scalp topography within P1 interval was not shown here because P1 on neither streaming nor bouncing trials showed positive amplitude, although both of them did show positive deflections. Note that there were no significant difference on C1, P1, and N1 amplitudes between streaming and bouncing trials.

Time-locking to the onset of the two discs (i.e., frame 1 onset; see Fig. 1) allowed us to examine the neural-activity patterns before the coincidence. Due to the two visual discs being placed 3.46° above the fixation cross (Fig. 1), these upper visual field stimuli elicited a typically negative C1 component peaking at 75–95 ms over the occipital scalp^56^ in both streaming and bouncing trials (Fig. 2A). The two-way ANOVA for this C1 component did not show a significant main effect of perceptual response [*F*(1, 17) = 2.32, *p* = 0.146, *η* ^2^_*p*_ = 0.12; streaming, −0.71 ± 0.27 μV (mean ± SE); bouncing, −0.40 ± 0.19 μV; Fig. 2B, upper row], and the main effect of electrode [*F*(5, 85) = 2.98, *p* = 0.062, *η* ^2^_*p*_ = 0.15] as well as the response x electrode interaction effect [*F*(5, 85) = 2.01, *p* = 0.155, *η* ^2^_*p*_ = 0.11] were also not significant. After C1, the occipitally distributed P1 component (with the maximal amplitude at 120–145 ms latency) also did not show a significant main effect of perceptual response [*F*(1, 17) = 0.47, *p* = 0.501, *η* ^2^_*p*_ = 0.03; streaming, −0.61 ± 0.44 μV; bouncing, −0.47 ± 0.54 μV], and neither the main effect of the electrode [*F*(5, 85) = 1.98, *p* = 0.161, *η* ^2^_*p*_ = 0.10] nor the response x electrode interaction effect [*F*(5, 85) = 1.00, *p* = 0.377, *η* ^2^_*p*_ = 0.06] was significant. Similarly, the subsequent N1 component peaking at 170–200 ms over occipital electrodes also did not reveal a significant modulation as a function of perceptual response [*F*(1, 17) = 2.13, *p* = 0.163, *η* ^2^_*p*_ = 0.11; streaming, −3.09 ± 0.49 μV; bouncing, −2.83 ± 0.50 μV; Fig. 2B, bottom row], and neither the main effect of the electrode [*F*(5, 85) = 2.85, *p* = 0.067, *η* ^2^_*p*_ = 0.14] nor the response x electrode interaction effect [*F*(5, 85) = 1.35, *p* = 0.271, *η* ^2^_*p*_ = 0.07] was significant. These results suggest that early visual-evoked ERPs before the coincidence of the two discs were not associated with distinct perceptual states in the visual streaming/bouncing display.

**Figure 3:**
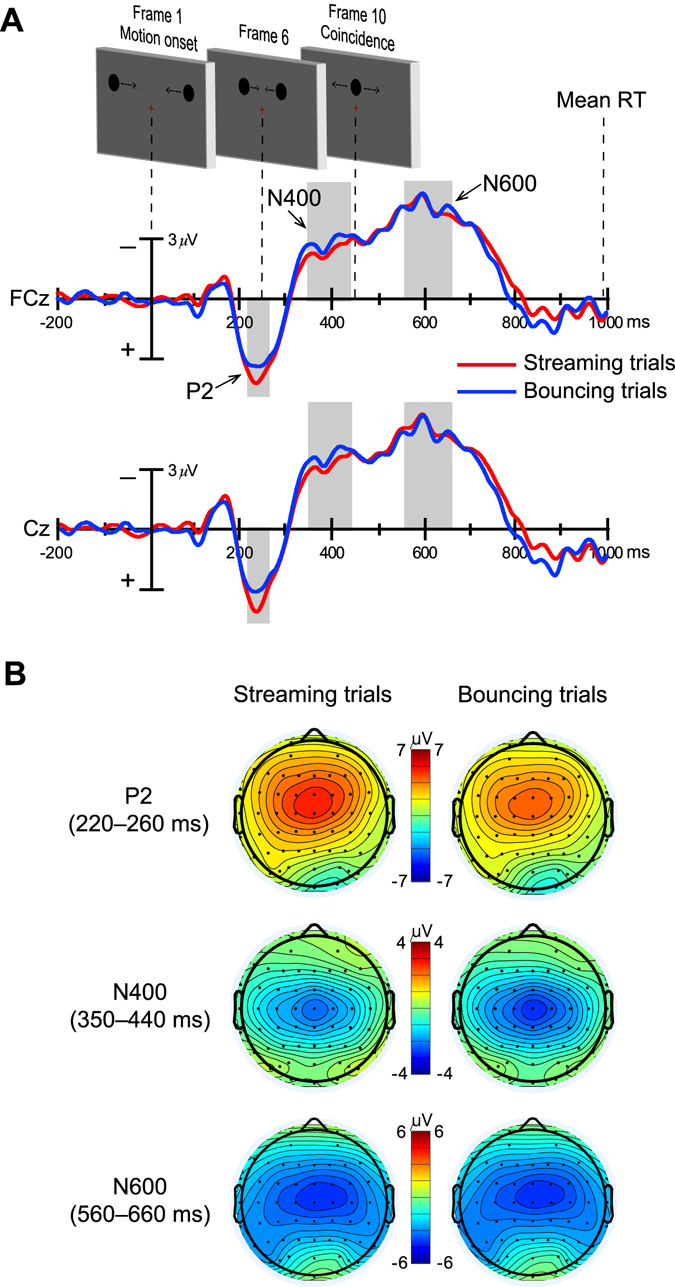
Grand-averaged ERPs associated with the streaming and bouncing percepts over anterior electrodes. (**A**) Three main ERP components over the anterior scalp, P2 (220–260 ms, shaded area, the same below), N400 (350–440 ms), and N600 component (560–660 ms), elicited by the visual streaming/bouncing motion display. Shaded areas show the time windows in which the three components were quantified respectively. Example waveforms shown here are from FCz and Cz electrodes. Key frames from stimuli motion sequences (see Fig. 1 for details) and mean RT (see Fig. 2 for details) are shown above the ERP waveforms. (**B**) Topographical distributions within the time intervals of P2, N400 and N600 for streaming trials and bouncing trials.

As shown in Fig. 3, motion of the two discs before their coincidence elicited an apparent positivity over the fronto-central scalp during 220–260 ms in both streaming and bouncing trials. This positivity is most likely to be a P2 component due to its time course and scalp topography^57^, and it is obvious that the P2 amplitude is larger on streaming trials than bouncing trials. Indeed, the two-way ANOVA showed a highly significant main effect of perceptual response [*F*(1, 17) = 10.19, *p* < 0.006, *η* ^2^_*p*_ = 0.38], with greater P2 amplitude on streaming trials (streaming, 3.75 ± 0.70 μV; bouncing, 3.10 ± 0.74 μV). The main effect of the electrode was also significant [*F*(5, 85) = 3.92, *p* < 0.05, *η* ^2^_*p*_ = 0.19]. Pair-wise comparisons for this main effect showed that P2 amplitude was larger on FCz than on FC2 (*p* < 0.03 after Bonferroni correction), and was larger on C1 and Cz than on C2 (*p*s < 0.05 after Bonferroni correction). The response x electrode interaction effect for P2 amplitude was not significant [*F*(5, 85) = 1.05, *p* = 0.379, *η* ^2^_*p*_ = 0.06]. Following the positivity of P2 component, there was also a negative deflection labeled as N400 component (Fig. 3A) over the fronto-central scalp (Fig. 3B, middle row) just before the coincidence of the two discs, which was maximal at 350–440 ms. The two-way ANOVA for this negativity also revealed significant modulation as a function of perceptual response [*F*(1, 17) = 6.19, *p* < 0.03, *η* ^2^_*p*_ = 0.27], with greater N400 amplitude for the bouncing trials (streaming, −2.58 ± 0.69 μV; bouncing, −2.98 ± 0.74 μV), and a significant main effect for electrodes [*F*(5, 85) = 5.55, *p* < 0.02, *η* ^2^_*p*_ = 0.25], with the N400 amplitude being larger on Cz than on C1 and C2 (*p*s < 0.03 after Bonferroni correction), as well as a nonsignificant response x electrode interaction effect [*F*(5, 85) = 0.48, *p* = 0.691, *η* ^2^_*p*_ = 0.03].

It is noteworthy that streaming and bouncing trials were separated based on subjects’ perceptual responses, and when ERP waveforms started to differ significantly in the P2 interval between streaming and bouncing trials, the two visual discs were still moving toward each other (Fig. 3A). In other words, even before the streaming/bouncing event actually occurred, the variations of neural activity (indexed by the fronto-central P2 component) have influenced the perceptual decision we would make after the streaming/bouncing event really occurred. In other words, the pre-coincidence difference on P2 component predicts the perceptual outcome of the visual streaming/bouncing display.

#### Post-coincidence P3 amplitudes dissociate streaming/bouncing responses

**Figure 4:**
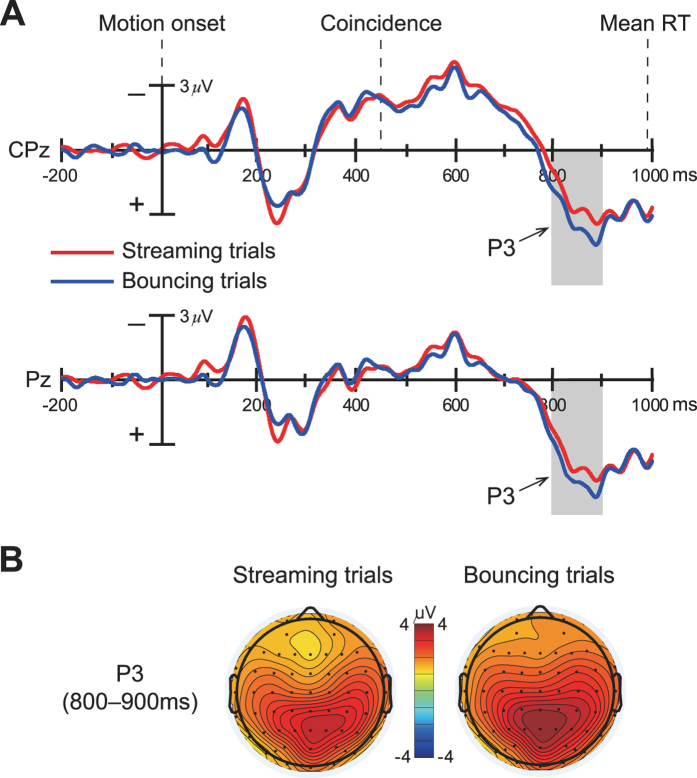
Grand-averaged ERP associated with the streaming and bouncing percepts over posterior electrodes. (**A**) P3 component (800–900 ms, shaded area) over parietal scalp elicited by the visual streaming/bouncing motion display. Shaded area shows the time window in which the P3 component was measured. Example waveforms shown here are from CPz and Pz electrodes. (**B**) Topographical distributions of P3 component for streaming trials and bouncing trials. Note that bouncing trials elicited larger P3 amplitude than streaming trials.

After the streaming/bouncing event occurred (i.e., the coincidence of the two discs), there was firstly a broad negativity with the maximal deflection during 560–660 ms (Fig. 3A) over fronto-central scalp (Fig. 3B, bottom row) for both streaming and bouncing trials, which was labeled as N600 component. The two-way ANOVA for the N600 amplitude revealed no significant difference between streaming and bouncing trials [*F*(1, 17) = 0.10, *p* = 0.753, *η* ^2^_*p*_ = 0.01; streaming, −4.67 ± 0.73 μV; bouncing, −4.60 ± 0.65 μV], and neither the main effects of the electrode [*F*(5, 85) = 3.56, *p* = 0.053, *η* ^2^_*p*_ = 0.17] nor the response x electrode interaction effect [*F*(5, 85) = 1.19, *p* = 0.312, *η* ^2^_*p*_ = 0.07] was significant. Following the N600 component, a late slow positivity extended over 800–900 ms for both the streaming and bouncing trials (Fig. 4A) is most likely to be the P3/P300 component given its broad amplitude distribution and parietal maximal scalp topography (Fig. 4B), which were associated with the detection of task-relevant events^58^. This slow positivity was quantified over 800–900 ms because both the maximal amplitude and detectable amplitude difference between the streaming and bouncing trials were included in this time window. Indeed, this obvious amplitude difference resulted in a significant main effect of perceptual response [*F*(1, 17) = 5.75, *p* < 0.03, *η* ^2^_*p*_ = 0.26], with greater P3 amplitude on bouncing trials (4.10 ± 0.70 μV) compared to streaming trials (3.28 ± 0.71 μV). The main effect of the electrode was also significant [*F*(5, 85) = 6.91, *p* < 0.006, *η* ^2^_*p*_ = 0.29], with the P3 amplitude being larger on Pz than on CP1 and CP2 (*p*s < 0.013 after Bonferroni correction). Finally, the response x electrode interaction effect for the P3 amplitude was not significant [*F*(5, 85) = 0.72, *p* = 0.485, *η* ^*2*^_*p*_ = 0.04]. These results indicated that the post-coincidence P3 amplitudes could also distinguish different perceptual outcomes in the visual streaming/bouncing display.

## Discussion

The present study investigated the neural basis of the processing of the classic streaming/bouncing motion display using high density event-related potential (ERP) recordings. The behavioral results showed no significant difference on RTs between the streaming and bouncing percepts, which was inconsistent with the findings of Sanabria *et al*. that found the dominant percept (i.e., streaming) in the visual streaming/bouncing display led to faster RTs^10^, although there was a tendency of faster RTs for streaming responses (streaming, 524 ± 20 ms; bouncing, 538 ± 21 ms) in the present study. A possible reason for this disparity might be that the existence of streaming/bouncing display with a salient sound at the moment of coincidence of the two discs [visual-auditory (VA) condition] in their experimental design influenced the response bias when participants perceived the purely visual streaming/bouncing display [visual-only (V) condition]. Therefore, when both VA and V conditions existed, salient sound in the VA condition would induce more bouncing percepts, which might result in hesitation when the subjects perceived streaming in the VA condition. Conversely, experience with the VA condition might also result in hesitation when subjects perceived bouncing in the V condition. Thus, the pattern that markedly faster RTs for bouncing percepts in the VA condition and faster RTs for streaming percepts in the V condition eventually occurred in study of Sanabria *et al*.^10^. However, the present study did not introduce a transient sound at the moment of coincidence, and so there was no response bias effect. Therefore, the results led to no substantial difference in RTs between streaming and bouncing percepts in the present study.

The ERP results showed a larger positive deflection on the frontal central P2 (220–260 ms) amplitude and N400 (350–440 ms) amplitude (with relatively smaller negative deflection on N400) before the coincidence for the streaming trials than bouncing trials. It is noteworthy that brain activities before motion or stimuli onset could bias subsequent perceptual results in ambiguous displays^38, 50, 59, 60^, which suggests that ongoing brain activities before stimulus-driven processes might contribute to how perceptual conflict is resolved by the human brain. The pre-coincidence differences in ERPs in the present study seem to make sense if we assume the motion sequences start at the moment of coincidence. Specifically, if the movement of the two discs started at the exact frame of their coincidence without any motion trajectory before coincidence, it is obvious that subjects in the behavioral task would perceive neither streaming nor bouncing but only two discs moving apart from the coincident point. Thus, the movement of the two discs before their coincidence actually served as a prerequisite for subsequent perceptual outcomes. Indeed, Grove *et al*. found that manipulating pre-coincidence trajectory switches (used to manipulate expectation) in streaming/bouncing display could significantly bias subsequent perceptual inferences^19^, which supports this viewpoint. It is also worth mentioning that the fMRI study that focused on the audiovisual bounce-inducing effect (ABE) conducted by Bushara *et al*. also investigated the purely visual streaming/bouncing display, but their data was time-locked to the coincidence of the two discs and found no difference in brain activity between the streaming and bouncing percepts^22^. When the fMRI results and our results showing a pre-coincidence difference on P2 and N400 components are combined, an inference can be drawn that subjects processed the motion information of two discs before their coincidence is an essential factor in influencing subsequent perceptual outcomes. In other words, the origin of ambiguity in visual streaming/bouncing processing may come from how subjects processed the motion information of the two discs before their coincidence. That is not to say other factors that occurred after or at the moment of the coincidence of the two discs are not important. In fact, a brief sound presented at the moment of coincidence could reverse the dominance of the streaming percept (i.e. ABE)^6^, and the post-coincidence trajectory duration was also found to be sufficient to bias subsequent perceptual inferences^17, 18^. Therefore, based on the present results and the discussion above, the pre-coincidence ERP differences found in the present study may reflect unconscious reduction of the ambiguity of streaming/bouncing display.

It has yet to be determined, however, that what aspects of perceptual process are reflected by the pre-coincidence positive deflection (i.e., larger positivity for the streaming percept than bouncing percept) that started ~200 ms before coincidence of the discs. Although the P2 component is typically thought as part of a cognitive matching system that compares sensory inputs with expectations derived from memory^57, 61^, and is involved in working memory^62^ and semantic processing^63^, its neurophysiological role has not been well characterized partly because it often overlaps with other adjacent ERPs such as N1, N2 and P3 while recording^64^. However, a recent study conducted by Shu *et al*. found that the P2 component is sensitive to depth perception, with larger P2 amplitude on three-dimensional (3D) than two-dimensional (2D) images (differences in physical properties between 3D and 2D images were minimized)^65^. Similarly, Liu *et al*. found that size perception was modulated by depth cues, with larger P2 amplitude on ball in upper than in lower visual field (the size of ball was always the same) for the 3D background condition but no difference for the 2D background condition^66^, indicating the P2 component is sensitive to depth information as well. If this is the case, the greater P2 amplitude on streaming than bouncing trials in the present study (Fig. 3) might be attributed to utilization of experience in a three-dimensional environment [objects in natural environment are seldom (but still possibly) aligned at the same depth plane] when perceiving visual streaming/bouncing display as Sekuler and Sekuler as well as Watanabe and Shimojo have proposed^5, 7, 17^ (see the Introduction section for details). In other words, when subjects considered the two moving discs as being at different depth planes (indexed by larger P2), they would perceive streaming after the coincidence, but when the two discs were thought to be at the same depth plane (indexed by smaller P2), the bouncing percept would be reported. Consistent with this account, Grove and Sakurai found streaming percepts increased as depth disparity between two discs increased in both the visual-auditory and visual-only streaming/bouncing display, although their focus was the audiovisual bounce-inducing effect (ABE)^67^. Similarly, Matsuno and Tomonaga investigated the visual streaming/bouncing display in chimpanzees and found streaming percepts increased when more depth cues were introduced^25^. Combined with the evidences and explanations above, the P2 amplitude difference between bouncing and streaming trials in the present study may reflect whether or not the subject perceived the two moving discs before their coincidence as being at the same depth plane. However, further research is still needed to examine whether the perceived depth between the two discs is the determinant factor for the visual streaming/bouncing illusion, because the present study did not test it directly.

As reported above, the pre-coincidence positive deflection that larger positivity was observed for the streaming percept than bouncing percept started ~200 ms before coincidence. However, the early visual-evoked ERPs (i.e., C1, P1 and N1 component) were almost identical between the streaming and bouncing trials (Fig. 2). It is easy to understand these results because these early visual-evoked ERPs were found to be very sensitive to changes in the physical properties of the stimuli^64^, whereas the stimuli (i.e., the motion of the two discs) inducing the streaming and bouncing percepts were always the same in our experiment. Thus, identical stimuli sequences elicited the similar early visual ERPs is exactly what would be expected. In contrast, as previous bistable perception studies demonstrated, due to the same physical inputs of stimuli, any changes in the electrophysiological response between mutually exclusive perceptions could therefore be ascribed to higher level perceptual or cognitive factors, rather than to factors relied on early sensory-input properties^48, 49^. This point of view fits the results of P2 in the present study and the prominent hypothesis that the P2 component represents part of a cognitive matching system that compares sensory inputs with expectations derived from memory^57^.

After the streaming/bouncing event occurred (i.e., coincidence of the two discs), the P3 amplitudes also dissociated streaming/bouncing responses, with greater P3 amplitude on bouncing trials than streaming trials (Fig. 4). Numerous previous studies have considered the P3 component to be an index of post-perceptual updates for working memory required to perform the perceptual-reporting task^68–71^. Overall, if no stimulus feature change is detected, the current mental pattern or “schema” of the stimulus representation will be sustained. If a new stimulus is detected, attentional processes will govern a change or “updating” of the current stimulus context, which is concomitant with larger P3 amplitude^64, 68, 72^. Meanwhile, the P3 component amplitude has been shown to increase for a more deviant (less probable) task-defined stimulus^73–77^. In the present behavioral results as well as previous studies of visual streaming/bouncing display, the streaming percept was dominant and occurred more frequently, whereas bouncing was the inferior percept and occurred occasionally^3–5^. Therefore, it is reasonable to infer that the streaming percept is subjectively the default state for subjects, which is considered as a highly probable perceptual outcome. Therefore, the bouncing percept is subjectively a novel perceptual outcome for subjects with a low probability. If that is the case, larger P3 amplitude for bouncing trials in the present study may reflect a novel bouncing percept with a low probability that triggered contextual updates for perceptual representation in subjects’ working memory. On the other hand, the P3 component in the present study seemed to appear so late that a time window of 800–900 ms was used to measure its amplitude. However, it makes sense because previous studies have shown that the latency of P3/P300 depends on the time required for stimuli classification, that is, P3 component should appear after classifying stimuli based on task requests^78–81^. Although ERP difference before streaming/bouncing event occurred had already influenced the subsequent perceptual outcomes in the present study, it is apparent that distinct perceptual outcomes must emerge after observing the occurrence of streaming/bouncing event. Specifically, classifying stimuli based on task requests (i.e., reporting either streaming or bouncing) in the present study actually started from the moment of the coincidence of the discs. Thus, if ERP waveforms were time-locked to the moment of the coincidence (450 ms), the time window of P3 component would become 350–450 ms, and along with its parietal maximal scalp topography (Fig. 4B), these characteristics were consistent with the results reported previously for typical P3/P300 component^58, 82^.

## Conclusion

In summary, the present study investigated the neural-activity patterns associated with the streaming and bouncing percepts to explore the origin of ambiguity and the neural basis of disambiguating perception during processing of the classic visual streaming/bouncing display. Interestingly, a frontal central positive deflection ~200 ms before the coincidence of the discs was found to be predictive of subsequent perceptual outcomes in the visual streaming/bouncing display. Moreover, P3 amplitudes ~400 ms after the coincidence of the two discs also dissociated streaming/bouncing responses. Base on previous studies of bistable perception that highlighted the role of high-level brain areas on perceptual interpretations^1, 44, 45^, and recent findings that the P2 component was sensitive to depth information^65, 66^, as well as the existing hypothesis that experience in a three-dimensional environment was involved in disambiguating the visual streaming/bouncing display^5, 7, 17, 19^, we conclude that the pre-coincidence frontal central positive difference between streaming and bouncing percepts in the present study may reflect whether or not the subject perceived the two moving discs before their coincidence as being at the same depth plane. Further studies are still needed to examine directly whether the perceived depth relationship between the two discs is the determinant factor for the visual streaming/bouncing display.
